# Variation in mouse chemical signals is genetically controlled and environmentally modulated

**DOI:** 10.1038/s41598-023-35450-8

**Published:** 2023-05-26

**Authors:** Romana Stopková, Tereza Matějková, Alica Dodoková, Pavel Talacko, Petr Zacek, Radislav Sedlacek, Jaroslav Piálek, Pavel Stopka

**Affiliations:** 1 Department of Zoology, Faculty of Science, BIOCEV, Charles University, Vestec, Prague, Czech Republic; 2 Czech Centre for Phenogenomics, Institute of Molecular Genetics of the Czech Academy of Sciences, Vestec, Czech Republic; 3 Research Facility Studenec, Institute of Vertebrate Biology, Czech Academy of Sciences, Brno, Czech Republic

**Keywords:** Evolutionary developmental biology, Sexual selection, Chemical ecology

## Abstract

In most mammals and particularly in mice, chemical communication relies on the detection of ethologically relevant fitness-related cues from other individuals. In mice, urine is the primary source of these signals, so we employed proteomics and metabolomics to identify key components of chemical signalling. We show that there is a correspondence between urinary volatiles and proteins in the representation of genetic background, sex and environment in two house mouse subspecies *Mus musculus musculus* and *M. m. domesticus*. We found that environment has a strong influence upon proteomic and metabolomic variation and that volatile mixtures better represent males while females have surprisingly more sex-biased proteins. Using machine learning and combined-omics techniques, we identified mixtures of metabolites and proteins that are associated with biological features.

## Introduction

All living organisms are constantly confronted with chemical signals from their environment and from other individuals. In mice, these signals often act upon innate^1^ or learned^2^ representations in the brain and yield behavioural responses that promote survival and fitness. For example, a male mouse will likely produce signals to advertise his fitness that would lead to avoidance behaviour in other males and to sexual attraction in females^3–5^. Some of these signals are species-specific or sub-specific, and used for peer-recognition^6,7^. Moreover, any individual, regardless of sex, will follow a cue that represents a favourite food item or avoid a cue indicating predators^8^. Most behaviour studies focus on the effect of a single or few compounds and proteins as signalling molecules. However, animals and their surrounding environments are more complex and instead of single or several studied compounds, most organisms also including bacteria^9^ and plants^10^ produce n-dimensional arrays of compounds. It is often the composition of these bouquets that induce behaviour and physiological responses in the receivers^11^. To make this puzzle even more complex, the response to the same cue might vary according to environmental factors. Thus, we asked whether biological traits such as sex and genetic background of an individual are manifested by proteomes or metabolomes and to what extent are these two sets linked or even correlated. This is important because sexual signals are known to prime sexually dimorphic circuits and striking sexually dimorphic sensory representations in the accessory olfactory bulb^12^ and the medial amygdala^13^, but a comprehensive view on chemical signals that may trigger these representations has been lacking. In general, we were interested in how sexuality is displayed in an organism for which the fitness-related olfactory cues are more important than the visual ones.

Mouse urine contains large quantities and a variety of molecules that serve as olfactory signals. They are detectable by chemosensory receptors of the major olfactory epithelia and/or vomeronasal organ (VNO)^14–22^. These signals yield diverse physiological responses in the receiver^13,23–31^ also when stimulated by selected non-volatile major urinary proteins (MUPs)^32,33^, short peptides^34,35^, and/or volatile organic compounds (VOCs)^36–38^. In mice, VOCs were considered as potent signals detectable by olfactory tissues^18,39^ whilst MUPs were mostly considered as transporters of these signals in their eight-stranded beta-barrels^37,40–47^ and thus shape the individual odour signatures^48^. However, various authors demonstrated that particular MUPs represent a signal on their own detectable by VNO^32,49–51^ and that some of these molecules including male-biased MUP20 (known as darcin) elicit complex innate behaviours including aggression^33^, mate recognition^52^ and learning^32^. However, since almost all previous studies concentrated only on MUPs, there is not yet any study showing the whole spectrum of urine proteins and volatiles that may also be involved in chemical communication, especially in wild living rodents.

Owing to their importance for male sexual signalling, MUPs are highly abundant and male-biased in the mouse urine^53–55^ where they protect and transport small volatile compounds in their eight-stranded beta barrel^40,56^ and also delay their release^57^. Interestingly, MUP patterns and the level of sexual dimorphism are sub-species specific^7,58,59^ making MUPs also important as candidate molecules in sub-species recognition^60–62^. Though females have less MUPs than males, these proteins are also involved in signalling of female sexual status because their concentration in the urine^29^ and vaginal secretions^63^ changes throughout the estrous cycle reaching the maxima during estrus. Similarly, social status affects the production of MUPs. This has been shown in wild derived *M. m. musculus* mice under laboratory conditions^28^ and in seminatural enclosures, where males doubled the excretion of MUPs after acquiring a territory and became socially dominant^64^. MUP quantity constitutes of up to 85% (or even more) of all proteins in the urine^65^, and thus these proteins may have distracted the attention from several hundreds of other important proteins that are involved in various homeostatic, metabolic and signalling functions.

To explore whether there are subspecific and sex-specific differences in the house mouse, we collected urine samples from several wild-derived strains representing two subspecies, *M. m. musculus* (MUS, 7 strains) and *M. m. domesticus* (DOM, 9 strains). Importantly, both groups of strains were kept in the same breeding facility. We aimed to detect major components of chemical signalling on the two levels of resolution – metabolomic and proteomic. Thus, we generated volatile metabolomes with head-space two-dimensional comprehensive gas chromatography and mass spectrometry and aliquots of the same samples were used in parallel for the analysis of proteomes with nLC-MS/MS. Subspecific and sex-specific differences served as a proxy for evolutionary changes due to natural or sexual selection. However, resulting profiles may as well be influenced by the natural environment, so we surveyed additional samples from wild caught *M. m. musculus* (wMUS) mice. Taken together, we analysed complete proteomes and metabolomes from the three mouse groups of each sex to provide new insights into the mouse chemical signalling.

## Results

### Data manipulation

The proteomic dataset contains a total of 958 protein identifications generated from 10 μl of urine of each sample and the resulting expression matrix was LFQ normalized (label-free quantification^66^). The same amount of urine (10 μl) was used to extract volatiles and the resulting data table contained a total of 2701 identifications based on unique masses and retention times. First, we reduced our datasets for singletons and doubletons such that only those molecules that were produced by at least three individuals from the same group (e.g. DOM males) were passed to further analyses. Final proteomic dataset thus contains 416 protein identifications. We did the same filtering for volatiles, however, volatile metabolomes are sensitive to false positives because the same molecules may naturally occur in animals but also in the air. To reduce the effect of false positives we removed all the molecules (i.e. rows) that were present only in blanks (i.e. samples of air from the labs where samples were processed). In the remaining set we have detected bimodal distribution, resulting from the mixture of two normal distributions. For these distributions overlap (see Methods), we calculated posterior p-value from normal-mixed models and if the p-value of belonging to blanks and samples was *p *< 0.05 (i.e. corresponding FD < 7.1) given rows were removed. This process corresponds to the Identity likelihood IL < 0.9 (see methods). IL is a useful tool to visualize whether given volatiles are likely to be characteristic of the studied groups. This dataset finally contained a total of 875 molecules and the whole set was quantile normalized. More than 54% of metabolome components in this study are structurally related aliphatic aldehydes and alcohols. The length of the carbon backbone of these molecules are typically C6–C8. The most abundant molecule is 2-hexenal (33,8%). 2-hexenal is part of so called ,,green odour” (GO), which is a mixture of eight aliphatic C6 aldehydes and C6 alcohols responsible for the smell of young leaves or freshly cut grass^1^. Several studies show high olfactory sensitivity of some mammals to GO^2^, including humans^3^ and indicate antidepressant^4^ and anxiolytic^2^ effects on mice.

### Sources of variation: sex, subspecies, or environment?

**Figure 1:**
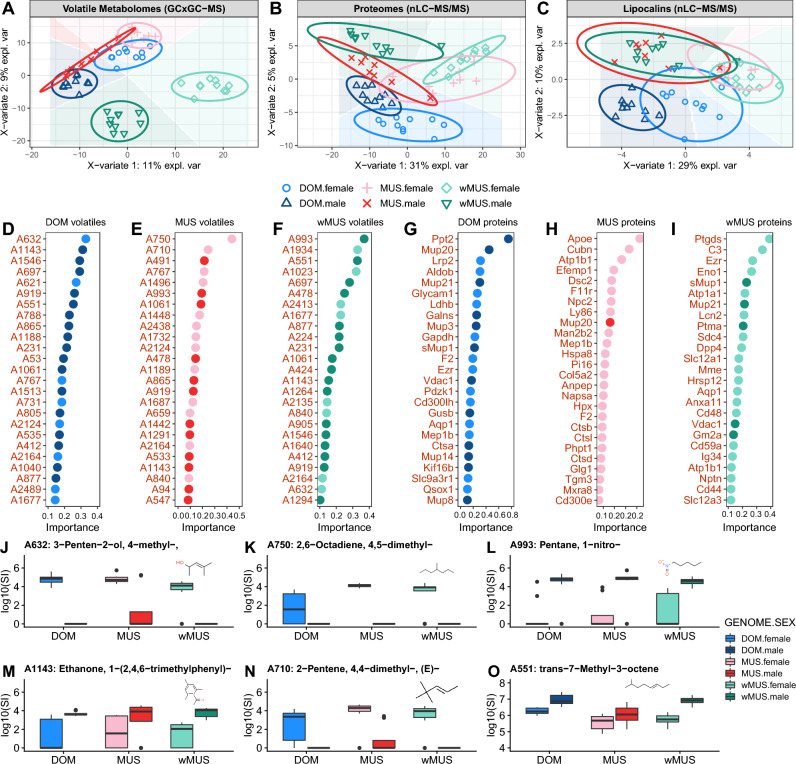
Proteomes and metabolomes are under genomic and environmental control. Discriminant analysis sPLS-DA revealed strong influence of environment upon metabolomes (**A**) and proteomes (**B**) in that the wild-derived mice wMUS are clearly separated from laboratory-bred MUS and DOM groups. However, lipocalins (**C**) are under genomic control, which is demonstrated by DOM males and females being separated from overlapping MUS and wMUS within each sex. Background prediction (polygons) is based on the Maximum distance method. To detect which are the proteins and volatiles that best represent each sex (**D**), we show with random forest for classification the top thirty sex-determining molecules that are ranked by the importance based on permuted Out of bag (OOB) scores (**D**–**I**). Top two examples of important volatiles and in each strain are in (**J**–**O**). Chemical structures are free downloads from https://www.chemspider.com. Colour code: darker colours are males lighter colours are females throughout.

To explore potential sources of variation in our data, we used Sparse Partial Least Squares Discriminant Analysis (sPLS-DA) for the fact that it has satisfying predictive performances in large datasets. In all the three comparisons in Fig. 1A–C, sex was the strongest factor influencing metabolome and proteome separation within the three mouse groups. We show in Fig. 1A that metabolomes of MUS and DOM of each sex are separated from each other. Since the MUS and DOM mice were kept for generations in the same conditions, this clear distinction between male vs. female metabolomes is most likely driven by genetic divergence between these two subspecies. However, so is separated wMUS of each sex from both the lab-bred MUS and DOM groups. This shows that urine metabolome of mouse is differentiated by subspecies, sex and environment. Thus, we predict that each component of the external environment such as food, microbiota^67^, plants, and naturally occurring air-born molecules may have direct influence upon metabolomic profiles and metabolite processing in an individual. Proteomic data in Fig. 1B show that sex is again the major driver of separation (31% of explained variation, x-variate 1). Due to the genomic differences, separation of MUS males from DOM males is clear and this is also true for females. However, similarly as in metabolomes, wMUS males (wMUS.male vs Other(s), Comp 2 (y-axis): Area Under Curve—AUC = 0.9761, *p *= 2.798e-06) and wMUS females (wMUS.female vs Other(s) AUC = 0.9543, *p *= 7.779e-06) are again separated (i.e. near-perfect discrimination) from all other groups, though in this scenario, wMUS and MUS are closer to each other than to DOM. This is reasonable evidence that all three factors (sex, group and environment) has an effect upon differentiation, though environment has a lower influence than in metabolomes. Next, we extracted only lipocalins with eight-stranded beta barrels and calycins with ten-stranded beta barrels from the whole dataset for their known transporting functions in chemical communication, reviewed in^68^. Here (Fig. 1C), MUS and wMUS males overlap and so do MUS and wMUS females. However, DOM females are less separated from DOM males and reach the lowest AUC scores (DOM.female vs Other(s), Comp 1 (x-axis) – AUC = 0.5630, *p *= 5.350e-01), but they are well separated from wMUS and MUS. This evidence also corroborates our previously reported finding that the level of sexual dimorphism in the expression of MUPs is higher in MUS than in DOM^7^. The major conclusion here is that lipocalin and calycin variation is driven by genetic differences (DOM vs. MUS) and not by environment (MUS vs. wMUS) while whole proteomes and metabolomes are environmentally modulated.

To detect which of these proteins and volatiles best predict the differences due to sex, we used random forest for classification (Fig. 1D-I). In Fig. 1J-O, we are showing examples of the most important compounds that best separate sex that have been detected with random forest. When looking at proteins, MUP20 (known as darcin) well represents sex in MUS (9th position) and DOM (2nd), but not in wMUS. For example, the 2nd position occupies the female-biased protein C3 (Power Law Global Error Model—PLGEM, FD_F-M_ = 20.6, P_wMUS_ = 0.02) that plays central roles in innate immunity and ENO1 on the 4th position (FD_F-M_ = 5.9, P_wMUS_ = 0.003) stimulates immunoglobulin production (extracted from UNIPROT functions, https://www.uniprot.org/). This discrepancy is likely caused by the fact that wMUS are direct offspring from wild-caught mice while MUS and DOM were bred and kept for generations in the same facility. The wild environment is immunologically more challenging than standard conditions of the mouse facility and thus wild mothers presumably transferred to their offspring their microbiota and an ‘immune memory’ which is influenced by the host microbiota^67,69^. This is also corroborated by the highly enriched and significant GO terms (FDR < 0.01) in wMUS (unlike MUS and DOM), which is dominated by the immune system process (proteins: CD48, C3, CD59A, CD44, SDC4, LCN2, DPP4, EZR). Furthermore, differences in the composition of microbial communities between lab-bred DOM and MUS, and wild mice have been previously studied in the same facility using similar mice and design^70^. They found that laboratory DOM and MUS have similar microbiota and that both are different from wMUS and wDOM. Their finding suggests that diverging microbial communities may contribute to proteomic and metabolomic variation.

### Levels of sexual dimorphism

**Figure 2:**
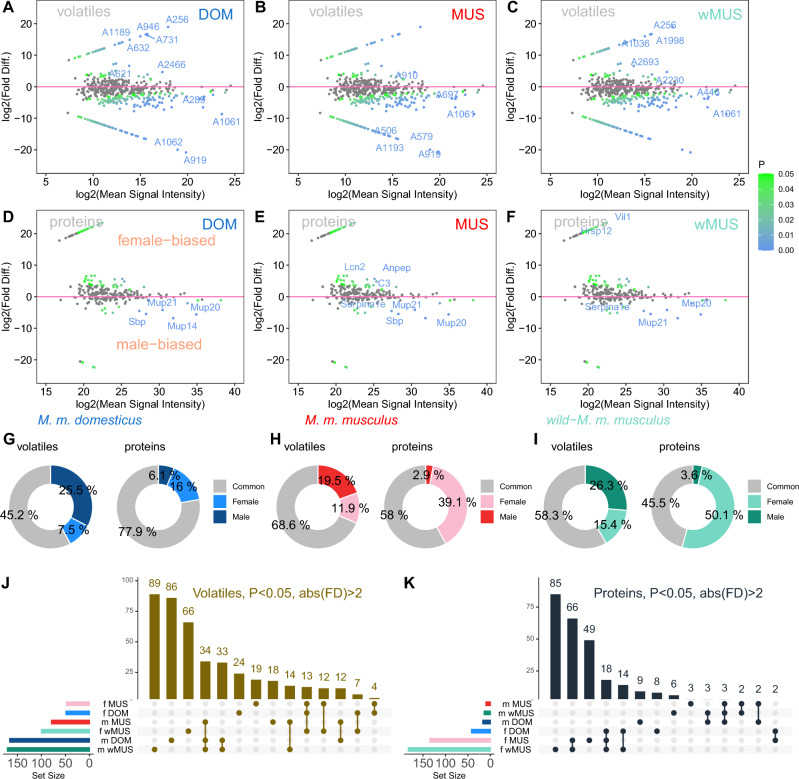
Sexually dimorphic molecules maintain sex- and strain-specific odour space. Differentially abundant volatiles (**A**–**C**) and proteins (**D**–**F**, *p *< 0.05, abs(FD > 2)) are scaled from green to blue but only top ten proteins and volatiles identified with ‘random forest’ as important are labelled with gene names or compound numbers. Above y = 0 are the female biased molecules while the male-biased are below the red line (y = 0). Next comparison involved significant sex-biased volatiles and proteins with *p *< 0.05 and abs(FD) > 2. In all three comparisons (**G**–**I**), males have more sex-biased volatiles while females have more sex-biased proteins. Though this pattern is significant in all the three groups, each group reveals sexuality by different volatiles and proteins (intersection plots in **J**–**K**). Abbreviations: abs() means an absolute value of; FD stands for fold difference.

In mice, mate selection does not rely only on females but both sexes are to some extent ‘choosy’^71,72^, so we asked an important question: how many proteins and volatiles are sexually dimorphic and do both subspecies use the same system of chemical signalling? To test the hypothesis that males and females have different urine chemical profiles and that the two subspecies might have evolved different systems of signalling, we used PLGEM models of differential expression to extract levels of sexual dimorphisms, and in combination with deep learning we aimed to identify most important (representative) molecules in the clouds of significant sex-biased data for each of the three mouse groups. In Fig. 2A-F, we show MA plots where volatiles (A–C) and proteins (D–F) are plotted as fold differences (FD) against the mean signal intensities and in log_2_ scale, Fig. 2A-F. Important proteins and volatiles that have been identified with Random forest and those that are ranked as top ~ 25 most important molecules (importance > 0.1) are labelled with gene or compound names. Reassuring message here is that most of the top molecules that were identified with deep learning were corroborated using the analysis of differential expression (e.g. MUP20 in DOM and MUS, MUP21 in wMUS).

The most interesting result that we report here Is that males produce larger variety of volatiles whilst females produce larger variety of proteins, while the total protein volume is substantially enriched in males. This divergence, which is consistent across the three studied mouse groups, is unlikely to have emerged by chance (Fisher’s exact test on counts: P_DOM_ = 2.248e-11, P_MUS_ < 2.2e-16, P_wMUS_ < 2.2e-16), Fig. 2G–I. In Fig. 2J–K we demonstrate using the intersection plots that sex identity is manifested by different molecules in each mouse group, or that those that are shared by all males (34 volatiles, 3 proteins) or by all females (13 volatiles, 18 proteins) are less common. This means that male and female odour spaces are dominated by molecules that have strain-biased expressions while those that are stereotypically produced in males or females regardless of the mouse origin are less common. Prime example of such shared molecule is MUP20 that is significantly male-biased in all the three studied mouse groups. Furthermore, MUP20 is not uniquely expressed only by males but is male-biased in all the three studied mouse groups. This means that previously reported pheromonal activities of darcin are driven by the expression differences rather than by sex-specific (unique) expressions.

### Integration of proteomes and metabolomes revealed new putative semio-chemicals

**Figure 3:**
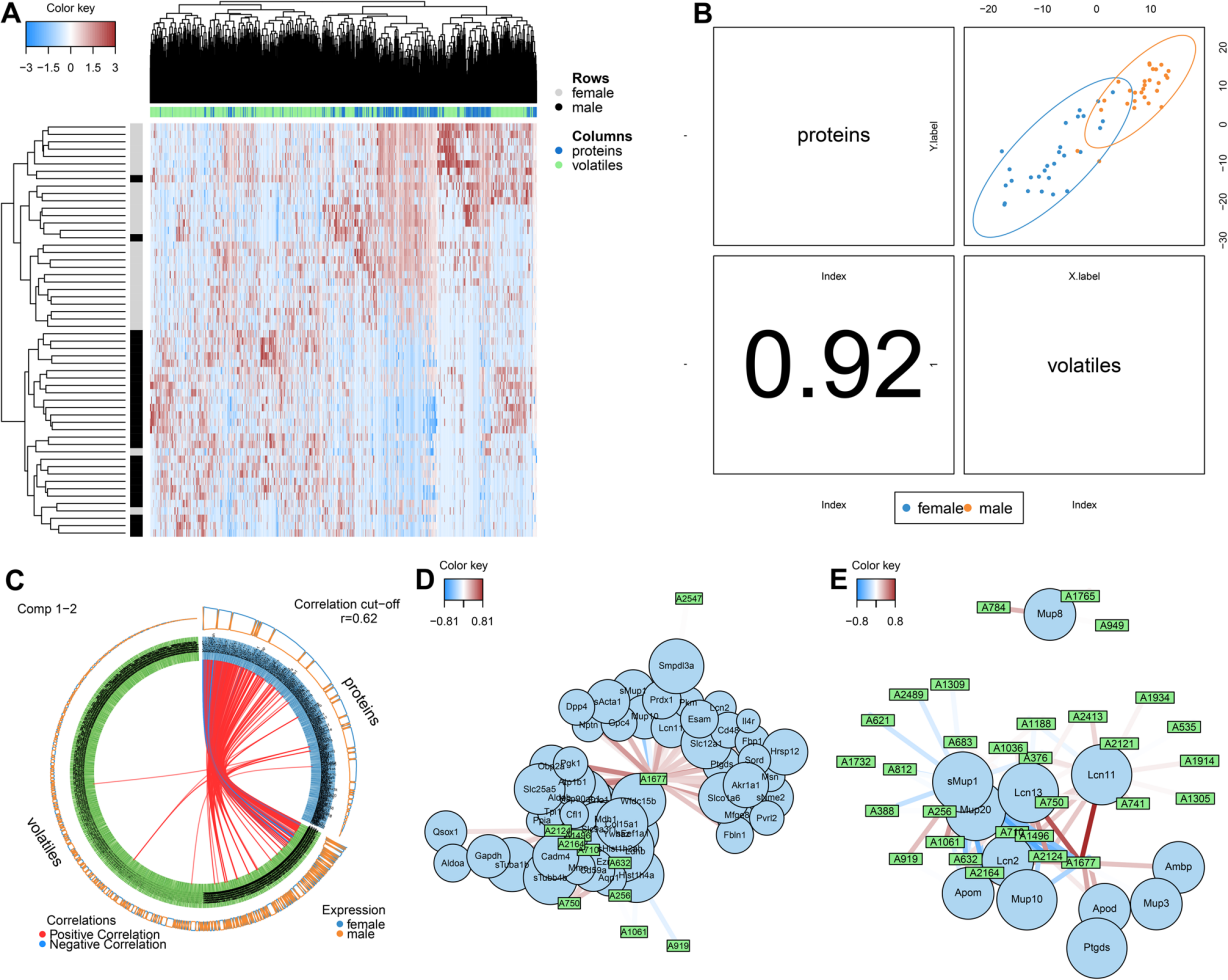
Integration of proteomes and metabolomes with correlation analysis revealed new potential interactions. Clustered Image Map (**A**) shows that correlated proteins and metabolites best explain sex differences across all individuals regardless strain. Males have more correlated volatiles while females have more correlated proteins. There is a positive correlation between proteins and volatiles at the component level (correlation = 0.92, *p *< 0.05) (**B**). This multi-omics molecular signature is mainly caused by the correlations between highly abundant proteins and volatiles (**C**), see the circular histograms. Stringent network analysis (correlation > 0.6) shows potential interactions between proteins and volatiles (**D**) and between (only) lipocalins and volatilesI).

In order to disentangle how proteomes and metabolomes interact, we used the discriminant analysis on blocks (i.e. block of proteomes and block of volatiles). First, we asked whether males and females from all the three groups have common and observable features that define maleness and femaleness. In Fig. 3A, we clearly see the blocks of proteins typical for females while blocks dominated by volatiles better represent males. We used the Area Under the Curve (AUC) to provide evidence that the discrimination is perfect in both dimensions (AUC1 vs. AUC2) in proteins and even better in volatiles (proteins: AUC1 = 0.9413, *p *= 1.429e-08, AUC2 = 0.9452, *p *= 1.071e-08; volatiles: AUC1 = 0.9796, *p *= 7.208e-10, AUC2 = 0.9821, *p *= 5.857e-10). A global overview of the correlation structure at the component level in Fig. 3B revealed strong correlation between proteomic and metabolomic data (r = 0.92), which offers an interpretation that both types of molecules represent sexuality in combinations. Figure 3C shows that the distribution of correlation coefficients (r > 0.65) is not random and that – based on the circular histograms – the most abundant proteins positively correlate with the most abundant volatiles. Thus, we extracted a network in Fig. 3D that represents the best correlations (r > 0.62) between proteins and volatiles.

The highest connectivity of a volatile to proteins and which has been identified as important by random forest (Fig. 1D,F) is typical for (A1677), which is 2(5H)-furanone, 5,5-dimethyl- (i.e. 5,5-dimethylfuran-2(5H)-one). It belongs to organic compounds known as butenolides. In our network, this sex-unbiased compound correlates with female-biased LCN2 and LCN11 and with male-biased MUP1 and MUP10. This connection is interesting because 2(5H)-furanone is a quorum-sensing molecule produced by fungi and bacteria to regulate bacterial growth and, for example, bovine OBPs scavenge this compound to prevent the bacterial growth thus turning to pathogens^73^. At the same time, LCN2 prevents bacterial growth by scavenging iron chelating bacterial siderophores in mice and humans^74^. Another important molecule (DOM in Fig. 1d, MUS in Fig. 1e) is A2124, which is (1,2,3,5,8,8a)-hexahydro-naphthalene, also known as dysoxylonene, a very hydrophobic molecule that belongs to sesquiterpenoids and in urine likely needs a protein transporter to enter aqueous environment. In our network, this compound also correlates with the female-biased protein LCN2, it is abundant in females of DOM, wMUS and MUS. This analysis also revealed high correlations between MUP20 and A919, which is 2-acetyl-3-thiazoline. This compound is highly similar to 2-s-butylthiazole, a natural ligand of darcin. However, in our data 2-acetyl-3-thiazoline better reveals maleness than 2-s-butylthiazole because it is unique in DOM and MUS males (~ 20 fold difference, *p *< 0.0001) and significantly male-biased in wMUS (~ eightfold difference, *p *< 0.0001). Moreover, the structures of 2-acetyl-3-thiazoline and 2-s-butylthiazole are so similar that they might have the same transporter darcin (MUP20). To obtain a further insight onto potential relationships between volatiles and proteins, we performed sPLS-DA on blocks of a complete volatile set and lipocalins without other proteins. The relationships described above are again supported in Fig. 3E but we also found some new and interesting associations. The prime example is MUP8 correlating with A784 which is 2-Methyl-1-nonene-3-yne. This compound has a plant/food origin and has high antimicrobial activity^75^. It is elevated in DOM males and slightly less in DOM females (FD = 2, *P *= 0.08 thus NS) whereas only a few MUS and wMUS individuals had this compound. The correlation between MUP8 and methyl-1-nonene-3-yne across all individuals and mouse groups (r = 0.62) is significant (*P *< 0.05). Although this approach yields interesting interactions between proteins and their potential ligands it is necessary to perform further binding experiments which are, however, beyond the scope of this study.

### Lipocalin code

**Figure 4:**
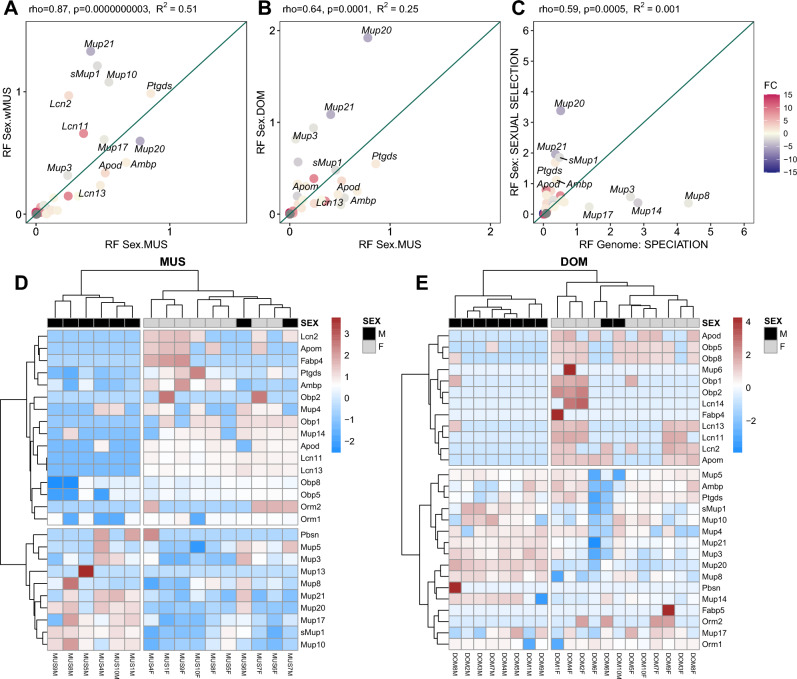
Combinatorial lipocalin code. Random forest (RF) plots of importance of particular proteins for sex separation: (**A**) wMUS against MUS; (**B**) DOM against MUS; in (**C**), the RF importance for separation of sexes is plotted against subspecies separation. Individual points are scaled from blue (male-biased) to red (female-biased). (**D**–**E**) Hierarchical clustering reveals the combinatorial nature of lipocalin abundances. In heatmaps, we demonstrate the relative contribution of proteins to sex separation using sPLS-DA (x-axis – individual names, y-axis – lipocalin gene names).

In this comparison, we performed random forest on a subset of proteins from the lipocalin family. First, we plotted random forest (RF) out-of-bag importance of individual proteins for sex separation in wMUS versus MUS (Fig. 4A) and found that Spearman rank correlation is high (rho = 0.86) and significant (*p *= 3e-10; R^2^ = 0.51). This is because they are genetically more alike and thus the same proteins are characteristic of sex separation. Similarly, we plotted the RF importance in DOM vs. MUS (Fig. 4B). As expected, correlation between DOM and MUS was lower (rho = 0.64; *p *= 0.0001; R^2^ = 0.25) than between MUS and wMUS, because of the genetic dissimilarity between the two subspecies. Plotting the RF importance for sex separation against the RF importance for subspecific separation (MUS, DOM) revealed very low correlation (rho = 0.59; *p *= 0.0005; R^2^ = 0.001), Fig. 4C. This diverging pattern provides evidence for the specialized nature of lipocalins where for example MUP20 and MUP21 reveal sex identity in all the studied groups while MUP14 and MUP8 abundances display subspecific status (see also Fig. 4D-E). Overall, MUP20 (darcin) is also the main driver of sexual dimorphism in our complete proteomic datasets. In heatmaps (Fig. 4D-E) only MUS and DOM are compared, because they were bred in the same facility. Here we demonstrate using sPLS-DA scores that there are more lipocalins in the urine than previously reported and that their variation is high. Hierarchical clustering corroborated that sex is a good though not an absolute predictor of lipocalin variation.

## Discussion

What is the most prominent feature of mouse chemical signals in the urine? Is it the volatiles or proteins? In contrast to other studies, we used higher volumes of urine (10 μl) for our label-free mass spectrometry directly from the samples and avoided using IPG strips and gels. Many gel-based MUP studies employed isoelectric focusing of proteins on IPG strips or gels with the range of isoelectric points pI 3.9–5.1 which only detect MUPs and not most other lipocalins with higher pI such as OBPs and LCNs that are more elevated in females. This approach often led to a focus on male MUPs as the main drivers of sexual dimorphisms, however, they are overrepresented in contrast to hundreds of other proteins that are present in the urine of females. We used the two house mouse subspecies DOM and MUS, which in Europe form a narrow hybrid zone running from Norway to the Black Sea^76,77^. We used sex-differences in the production of compounds as a proxy for sexual selection while subspecies differences were a proxy for speciation. We had yet another set of wMUS animals that served to find out whether the combination of environment and hygienic status (wild vs. lab-bred) influences urinary profiles.

For the first time, we show that female mice have a variety of female-biased lipocalins in their urine and that some of these lipocalins were previously detected only in mucosal secretions of eyes^78^, nose^79–82^, oral cavity^83^, and vagina^63,84^. MUPs are highly expressed in the liver^85^ and it has been repeatedly demonstrated that they are excreted to the urine and deposited as urine marks thus slowly releasing their ligands (VOCs). Mouse OBPs are not expressed in the liver^86^, however they are abundant in vagina where they are co-expressed with other detected proteins including LCN11, LCN2, MUP9, darcin, and other lipocalin members. They are upregulated during estrus and metestrus and they drop to lower levels in proestrus^63^. Thus, it is likely that female glands and reproductive organs produce some of the proteins detected in their urine, which reflects their reproductive state. This corroborates our previous studies which demonstrated that the abundance of female MUPs in the urine correlates with the estrous cycle in the laboratory^29^ and wMUS^28^ mice, reviewed in^68^.

Taking a broader view, we touched a fundamental question in biology, namely how is sexuality signalled^87^ in animals that primarily depend on olfactory cues^88,89^ and whether a single pheromone or a mixture of compounds may potentially serve to prime social and reproductive behaviours of the receiver. In mammals, sexuality is often maintained by sexual dimorphisms where some components evolved to display sexual traits that are specifically processed in the brain^12,13,90^ while others are the consequences of sex-biased metabolic processing^36^ and immune defence^91,92^. Not all proteins and compounds that are sex-specific are involved in chemical signalling. But from our data and other studies we can see that it is likely that a small effect of many molecules, rather than a strong effect of few, is characteristic for mouse chemical signals, similarly as in mole rat perioral secretions^93^. In our data, sexuality is well displayed via lipocalins (e.g. MUPs, OBPs, LCNs) that are known for their roles in chemical communication and by several volatiles that have been studied in many laboratories (e.g. SBT, farnesenes, pyrazines etc.) and species, for example the mole rats^93^. However, the majority of proteins and volatiles in our data have not been previously studied in the context of sexual signalling. Of course, it would be best to test each of the detected compounds in individual behavioural setups, but this is practically impossible. Another option, presented in this paper is a pre-selection based on the searches of compounds that have correlated patterns and thus may have the potential to represent biological features such as sex, sub-species and hygienic status of an individual. If a volatile is too hydrophobic, it needs a protein transporter that can help the ligand to enter aqueous environment (i.e., urine). Thus, it is reasonable to expect that proteins and volatiles will to some extent be correlated and this is exactly what our study shows. Volatiles are correlated with proteins but only few are organized in larger networks of proteins and their potential ligands. When these correlations are extracted, we can see putative relationships between combinations of proteins and ligands such as MUP20 and 2-acetyl-3-thiazoline as well as the new putative pairs (e.g. LCN11 and 2(5H)-furanone). We do realize that correlation is not the same as causation, but this approach may lead to new set of hypotheses based on the complexity of the molecular profiles indicated by this study..

To conclude, we used deep learning and data integration to identify in the urine metabolome and proteome of mice, molecules that are sex- and subspecies- specific, and are likely involved in chemical signalling. We have also shown for the first time that sexuality is displayed by at least 26 different lipocalins and calycins (12–16 are female biased) and not just by male-biased MUPs. However, the number of shared peptides in this group of proteins exposes a need for absolute quantification of these proteins, based on unbiased methods. Furthermore, striking differences in the abundance of sex-biased molecules between DOM and MUS revealed that there was a strong selection on systems of sexual signalling during the speciation of DOM and MUS mice.

## Materials and methods

### Ethical standards

All animal procedures were carried out in strict accordance with the law of the Czech Republic paragraph 17 no. 246/1992. Handling of wild MUS was approved by the local ethics committee of the Faculty of Science, Charles University in accordance with accreditation no. 27335/2013-17214. Wild-derived mice were kept in the breeding facility of the Institute of Vertebrate Biology in Studenec (authorized by the Ministry of Agriculture 61974/2017-MZE-17214). The following strains represented MUS (Armenia: MAM, Czechia: MCZ and PWK, Georgia: MGA, Poland: MPB, Bulgaria: SOK and SVEN) and DOM (Algeria: BZO, Austria: BING and URG, Cyprus: DCA and DCP, Denmark: DDO, Italy: DJO, Portugal: SAGR and SOAL) (for details see^94–96^, and https://housemice.cz/en/strains/). This study was performed and reported in accordance with ARRIVE guidelines (https://arriveguidelines.org).

### Subjects, housing conditions and experimental design

**Figure 5:**
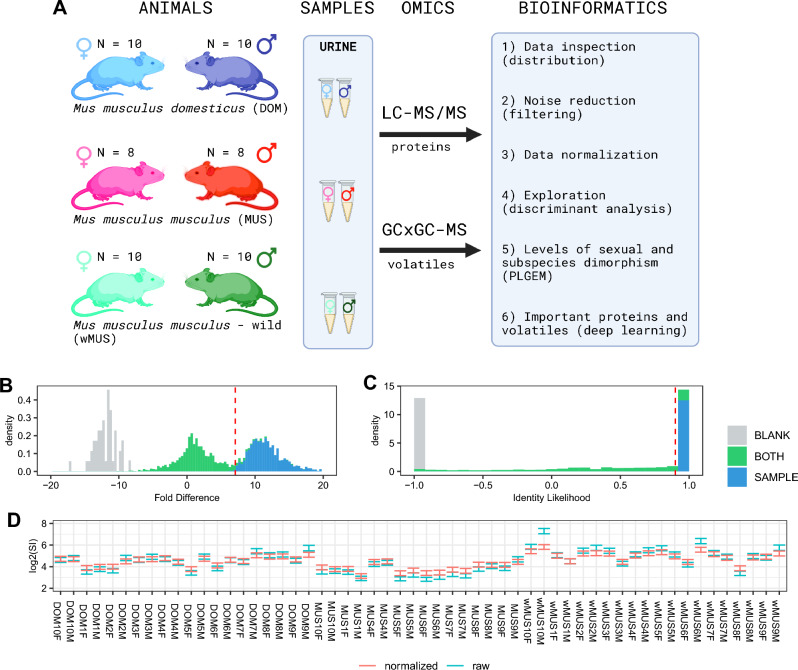
Experiment design, filtering, and normalization. We used mouse urine repeatedly sampled from individuals of each sex from the three groups – laboratory-bred DOM and MUS, and wild wMUS (**A)**. We focused on the analysis of their urine proteins and volatiles. We excluded volatiles that occurred only in blanks (**B**, grey bars), whilst those that occurred in blanks and samples (green) were selected based on the posterior p-values of the mixed-normal model (see methods). Those that had *p *< 0.05 (i.e. corresponding FD < 7.1) of belonging to blanks and samples were removed (red line); this corresponds to the Identity likelihood IL < 0.9 (**C**). Remaining compounds (N = 875) were considered as relevant because they only occurred in samples or in significantly higher quantities in samples than in blanks (FD > 7.1. Quantile normalisation yielded reasonably low variation in signal intensities (SI) between samples (**D**). FD stands for fold difference. Tubes, mouse pics and chemical structures (**A**) were created by the authors with BioRender.com.

In this experiment (Fig. 5A), we used a total of 56 mice from the three groups: wild *Mus musculus musculus* (wMUS: direct offspring from wild-caught mice, 10 pairs), laboratory *M. m. musculus* (MUS: generations G3-G60 of lab-bred mice, 8 pairs) and laboratory *M. m. domesticus* (DOM: generations G1-G67 of lab-bred mice, 10 pairs). All individuals had the same diet (ST1 pellets, Velaz, Prague, Czech Republic) and water ad libitum and were kept under stable conditions (i.e. 14:10 h, D:N, temperature t = 23 °C). We collected their urine four to six times to overcome potential differences in dilutions. Final volume of urine was between 25 and 100 μl per mouse and all samples were frozen (− 80 °C) before further analyses. These samples were measured with GCxGC-MS/MS and in parallel additional 10 μl of samples were used for the nLC-MS/MS analyses of proteins (see below). None of the animals was euthanized or sacrificed during the sampling. We only manipulated individuals such that they urinated to a collection tube and then they were returned to their cage.

### Detection of volatile metabolites

Urine volatiles were sampled using Headspace Solid Phase Micro Extraction (HS SPME) on fiber (DVB/CAR/PDMS_grey; Supelco, USA). Samples were incubated for 5 min at 55 °C prior to the extraction. The extraction was carried out for 10 min. The volatiles were analyzed using two-dimensional comprehensive gas chromatography with mass detection (GCxGC-MS; Pegasus 4D, Leco Corporation, USA). A combination of mid-polar and non-polar separation columns was used for the separation: Primary column: SLB-IL60 (30 m × 0.25 mm, SigmaAldrich, USA); Secondary column Rxi-5sil MS (1.4 m × 0.25 mm, Restek, Australia). Other parameters were set as follows: inlet temperature 270 °C, spitless mode, constant He flow 1 ml/min, modulation time 4 s (hot pulse 0.6 s), modulation temperature offset with respect to the secondary oven 15 °C. The temperature program applied on the primary oven: 50 °C (hold 1 min), then increase (10 °C/min) to 320 °C (hold 3 min). The temperature offset applied on the secondary column was + 5 °C. Transferline temperature was held on 250 °C. The mass detector was equipped with an EI ion source and TOF analyzer enabling unite mass resolution. The scanned mass range was 30–500 m*/z*. The ion source chamber was held on 250 °C. LECO’s ChromaTOF v4.5 was employed to control the instrument and for data processing. Selected compounds were identified by matching their mass spectra with a library of mass spectra (NIST MS 2.2, USA).

### Analysis of volatile metabolites

We generated histograms of data distribution and removed all the rows with compounds that occurred only in blanks and not in samples. The resulting distribution is bi-modal with compounds that occurred only in samples and in samples and blanks, Fig. 5B. To decide which compounds are biologically relevant, we used ‘mixtools’^97^ routine which calculates the posterior probability for the identity to either of the two peaks within the mixture of two overlapping normal distributions. We excluded all the compounds, which had the identity to blanks and samples with *p *< 0.05, Fig. 5C. To visualize the likelihood of identity to either of the two peaks, we used a simple index of identity LI = (sample – blank)/(sample + blank) ranging from − 1 to1 so all the remaining ‘biologically relevant’ compounds had LI > 0.9 (Fig. 5C). Next, we used a normalization based upon quantiles, which normalizes a matrix of peak areas (i.e. intensities) with the function normalize.quantiles of the ‘preprocessCore’ package in R software^98^, visualised in Fig. 5D. To extract p-values of differentially abundant compounds, we used the Power Law Global Error Model – PLGEM^99^ similarly as in the analysis of proteomes (see below).

### Protein Digestion and nLC-MS/MS Analysis

All protein samples were cold-acetone precipitated and centrifuged at 14,000 rcf for 10 min at 0 °C. This was followed by a re-suspension of dried pellets in the digestion buffer (1% SDC, 100 mM TEAB – pH = 8.5). The protein concentration of each lysate was determined using the BCA assay kit (Fisher Scientific). Cysteines in 20 μg of proteins were reduced with a final concentration of 5 mM TCEP (60° C for 60 min) and blocked with10mM MMTS (i.e. S-methyl methanethiosulfonate, 10 min room temperature). Samples were cleaved with trypsin (1 ug of trypsin per sample) in 37 °C overnight. Peptides were desalted on a Michrom C18 column. Nano Reversed phase columns were used (EASY-Spray column, 50 cm × 75 µm ID, PepMap C18, 2 µm particles, 100 Å pore size**)**. Eluting peptide cations were converted to gas-phase ions by electrospray ionization and analysed on a Thermo Orbitrap Fusion (Q-OT-qIT, Thermo) with the same parameters as described in^78,79,83^.

### Proteomic analysis

LC–MS data were pre-processed with MaxQuant software (version 1.6.34)^66^. The false discovery rate (FDR) was set to 1% for both proteins and peptides and we specified a minimum peptide length of seven amino acids. The Andromeda search engine was used for the MS/MS spectra mapping against our modified Uniprot *Mus musculus* database (downloaded in June, 2015), containing 44,900 entries. We modified our databases such that all MUP and OBP sequences were removed and instead of them we have added a complete list of MUPs from Ensembl database, and OBPs from NCBI (sensu—citation^86^). Next, we added some Tremble sequences that were missing in Uniprot, for example KLKs, BPIs, SPINKs, SCGB/ABPs, and LCNs. We provide this dataset in FASTA format as Supplementary dataset 1. Enzyme specificity was set as C-terminal to Arg and Lys, also allowing cleavage at proline bonds^100^ and a maximum of two missed cleavages. Dithiomethylation of cysteine was selected as fixed modification and N-terminal protein acetylation and methionine oxidation as variable modifications. The `match between runs` feature of MaxQuant was used to transfer identifications to other LC–MS/MS runs based on their masses and retention time (maximum deviation 0.7 min). Quantifications were performed using the label-free algorithms^66^ with a combination of unique and razor peptides. All subsequent analyses were performed in R software^98^. To check that the data distribution conforms to the same type of distribution after normalization, we used ‘mixtools’^97^. Second, we used the Power Law Global Error Model—PLGEM^99^ to detect differentially expressed / abundant proteins using the functions plgem.fit and plgem-stn^97^. To detect the importance of significant proteins in separation between males and females we used Random Forest for Classification^101^ within the R software^98^. All plots and figures were generated in R using ggplot2^102^. R software is distributed under the terms of the GNU General Public License. Copies of both versions 2 and 3 of the license can be found at https://www.R-project.org/Licenses/. Original and LC–MS/MS and GCxGC/MS tables are provided in Supplementary dataset 2.

## Supplementary Information

Supplementary Information 1.

Supplementary Information 2.

## Data Availability

The mass spectrometry proteomics data have been deposited to the ProteomeXchange Consortium via the PRIDE partner repository with the dataset identifier PXD037086 and 10.6019/PXD037086. Resulting tables are available as supplementary data. Metabolomics data have been deposited to the EMBL-EBI MetaboLights database (10.1093/nar/gkz1019, PMID: 31691833) with the identifier MTBLS7422. The complete dataset can be accessed here https://www.ebi.ac.uk/metabolights/MTBLS7422.
